# Syphilis and cerebral stroke: a case report and systematic review

**DOI:** 10.1186/s42466-026-00510-7

**Published:** 2026-08-03

**Authors:** Judith N. Wagner, Matthias Klein, Susanne Dyckhoff-Shen

**Affiliations:** 1 University Hospital Essen, University Duisburg-Essen, Essen, Germany; 2 Department of Neurology, LMU University Hospital, LMU Munich, Munich, Germany

**Keywords:** Stroke, Neurosyphilis, Treponema pallidum

## Abstract

**Background:**

Syphilis is a multifaceted disease caused by *Treponema pallidum.* It has re-emerged as a public health concern and represents a potentially treatable cause of cerebrovascular disease. Neurosyphilis (NS) can involve the central nervous system at any stage of infection, with meningovascular syphilis leading to ischemic or, less frequently, hemorrhagic stroke. Diagnosis remains challenging due to heterogeneous clinical presentations and challenging serological diagnostics.

**Methods:**

We report a case of ischemic stroke as the first manifestation of syphilis and performed a systematic review of the literature on syphilis-associated stroke. A structured search of PubMed, the COCHRANE Library, and secondary sources was conducted for studies published from 1972 onward.

**Results:**

The reported case describes a 44-year-old HIV-negative man presenting with ischemic stroke due to presumed small-vessel meningovascular syphilis, supported by CSF pleocytosis and positive treponemal serology in serum and CSF. The patient improved substantially following ceftriaxone therapy. The systematic review identified 98 relevant publications, including 73 individual cases and 36 cohort studies. Stroke frequently led to the initial diagnosis of syphilis and predominantly affected younger male patients. Ischemic stroke was the most common presentation, mainly due to large-vessel disease. Penicillin was the most frequently used therapy, with ceftriaxone as an effective alternative.

**Discussion:**

Syphilis is an important and underrecognized risk factor for stroke, particularly in younger patients. NS should be considered in cases of cryptogenic stroke, and standardized diagnostic and therapeutic approaches are needed to improve recognition and outcomes. Few data on the specific stroke treatment in syphilis exist.

## Introduction

Syphilis is an infectious disease caused by the bacterium Treponema pallidum (T. pallidum). It is usually transmitted via sexual intercourse and often coexists with other sexually transmitted diseases such as HIV. Due to decreasing use of safer sex practices, syphilis prevalence has continually increased during the past decade [1]. It is thus a disease of public health concern.

The disease manifests in various stages: chancres at the site of contact with the infectious lesion in primary syphilis and skin manifestations (rash, mucosal lesions), lymphadenopathy and involvement of inner organs in secondary syphilis [1]. Although neurological symptoms may present in any stage, they are most typical of tertiary syphilis, when they manifest as meningovascular or parenchymatous (general paresis, tabes dorsalis) disease. However, some patients with syphilis and cerebrospinal fluid (CSF) abnormalities are asymptomatic, so-called latent neurosyphilis (NS) [2].

Meningovascular syphilis can result in ischemic and hemorrhagic cerebral strokes. They are caused by a chronic inflammatory arteriopathy leading to luminal obstruction and thrombus formation due to endothelial dysfunction [3]. While the latter is the more common cause of stroke in inflammation of large and medium-sized arteries (so-called “Heubner endarteritis”), the former typically occurs in the “Nissl-Alzheimer” type, a small vessel syphilitic arteritis. Embolic cerebral strokes may also be due to syphilitic cardiac or aortic involvement [4, 5].

HIV and Treponema infection often coincide and there is evidence of an increased risk of stroke in HIV patients with NS comorbidity [6, 7]. HIV infection itself seems to be a risk factor for stroke, particularly if the CD4 count is low (i.e. < 200 cells/µl) [8].

Serology is the mainstay of diagnosis. While specific treponemal tests such as fluorescent treponemal antibody absorption (FTA-ABS), Treponema pallidum particle agglutination assay (TPPA), and microhemagglutination assay for Treponema pallidum antibodies (MHA-TP) confirm exposure of the immune system to Treponema pallidum, they cannot differentiate between a previous or current infection. Reactions not specific to T. pallidum (e.g. venereal disease research laboratory test (VDRL), rapid plasma reagin test (RPR)) are used to fill this gap and as a parameter to monitor disease activity and response to therapy [2]. These tests can be performed on both serum and CSF. However, the sensitivity of these non-specific tests reach only 30–85% [9–11]. The diagnosis of NS rests on a combination of clinical findings, inflammatory CSF changes, and detection of intrathecal synthesis of T. pallidum-specific antibodies [12, 13].

Penicillin remains the therapy of choice for NS, while Ceftriaxone may be used as well. Few data exist on the specific acute and prophylactic treatment of syphilis-associated cerebral vascular disease.

We report on a patient who presented with ischemic stroke as the first manifestation of syphilis and the results of a systematic review on stroke and syphilis.

## Methods

For the case report, patient data were retrieved retrospectively from the electronic patient files at Evangelisches Klinikum Gelsenkirchen. Patient consent for publication was obtained.

For the literature review, we performed a MEDLINE literature search using PubMed, the COCHRANE Library and secondary literature search to identify all reports published between January 1, 1972, and December 31, 2025. The starting date was set to match the introduction of the computed tomography (CT) scan into clinical routine. The PubMed search terms were treponema OR "syphilis" OR "neurosyphilis" OR "lues" AND ("meningovascular" OR "stroke" OR "ischemic stroke" OR "imaging" OR “infarct*”), the COCHRANE search term was “stroke AND syphilis”.

Titles and abstracts of the reports obtained were screened for inclusion in the review using the following criteria: population with a diagnosis of syphilis and stroke (ischemic or hemorrhagic stroke or transitory ischemic attack), and sufficient data to evaluate the causality of treponemal infection for the stroke. For the diagnosis of NS, we required proof of intrathecal inflammation (raised CSF cell count, positive CSF VDRL or RPR, or corresponding autopsy findings).

Articles published in languages other than English, German, or Spanish as well as duplicate studies, preclinical studies, editorials and reviews were excluded. No further exclusion criteria applied. Included were all case reports, case series, retrospective and prospective observational studies, and randomized controlled trials. A secondary search for other relevant articles was performed in the articles included after full-text analysis as well as in reviews on the topic.

The study was approved by the ethics committee Westfalen-Lippe, Münster, Germany (reference number 2023–436-f-S).

## Case report

**Fig. 1:**
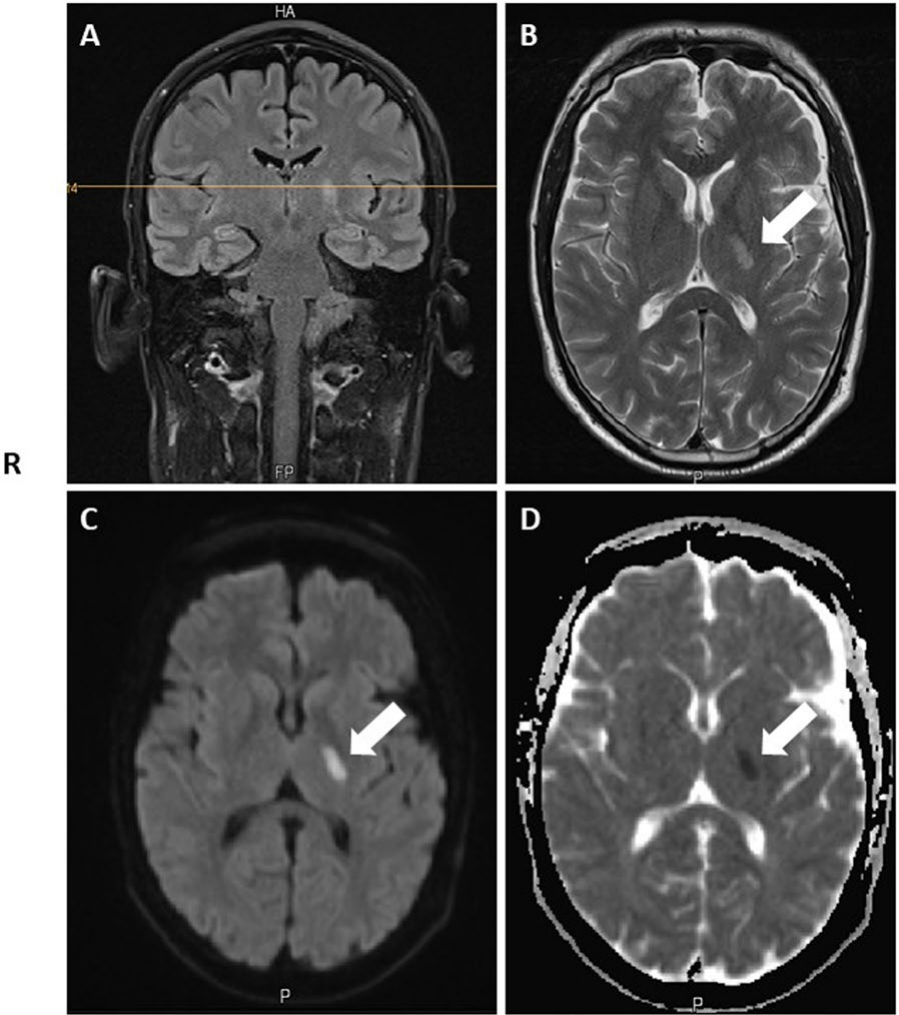
Cerebral MRT of reported patient. Coronar FLAIR (**A**), transversal T2 (**B**), diffusion weighted (**C**), and ADC map (**D**) imaging reveal a lacunar infarction in the left internal capsule (arrow). The horizontal line in (**A**) denotes the slice position of (**B**-**D**)

We report on a 44-year-old male HIV-negative patient who had sex with men without relevant pre-conditions who presented with a fluctuating right-sided hemiparesis and dysarthria. He had no peripheral nerve palsies. Cerebral MRI showed an ischemic lesion in the left internal capsule (Fig. 1). Ultrasound revealed minimal carotid atherosclerosis. There were no intracranial arterial stenosis or string of beads sign on CT angiography. 24h-ECG showed continuous sinus rhythm. Other cardiac sources of cerebral emboli were excluded via transesophageal echocardiography. Blood pressure was normal throughout.

To investigate for rarer causes of juvenile stroke, a lumbar puncture was perfomed. The cerebrospinal fluid (CSF) showed a monomorphic pleocytosis (178/µl, 100% monomorphic leukocytes), slightly raised total protein 54mg/dl, and normal lactate (19.2mg/dl). T. pallidum IgG in serum and CSF returned positive, a positive serum-VDRL test suggested active disease (Table 1). Bands of anti-treponemal IgG in the CSF were more intense than in the serum (Tp47: 3.0 vs 2.5; TmpA: 2.3 vs 2.0; Tp17: 3.8 vs 3.0), but CSF/serum antibody index was not calculated. IgM-type antibodies against T. pallidum were negative. These results are compatible with active syphilis and very suggestive of NS. However, as no markers of disease activity were measured in the CSF, the patient does not fulfill the criteria of definitive NS. Hence, we diagnosed likely NS. The patient was treated with a 15-day-regimen of ceftriaxone, which we had started empirically before receiving the positive T. pallidum serology.

**Table 1:** Serological findings (serum) in the reported patient. WB = Western Blot

VDRL	1:16
**Treponema pallidum IgM (WB)**	Negative
Tp47	-
TmpA	-
Tp257	-
Tp453	-
Tp17	-
Tp15	-
**Treponema pallidum IgG (WB)**	Positive
Tp47	+ + +
TmpA	+ + +
Tp257	+ + +
Tp453	+ + +
Tp17	+ + +
Tp15	+ + +

We made the diagnosis of cerebral infarction due to small vessel arteritis. The patient was discharged to a rehabilitation unit with a residual moderate hemiparesis on the right. On his last follow-up, he had recovered almost completely (mRS 1 after 255 days).

## Results systematic review

**Fig. 2:**
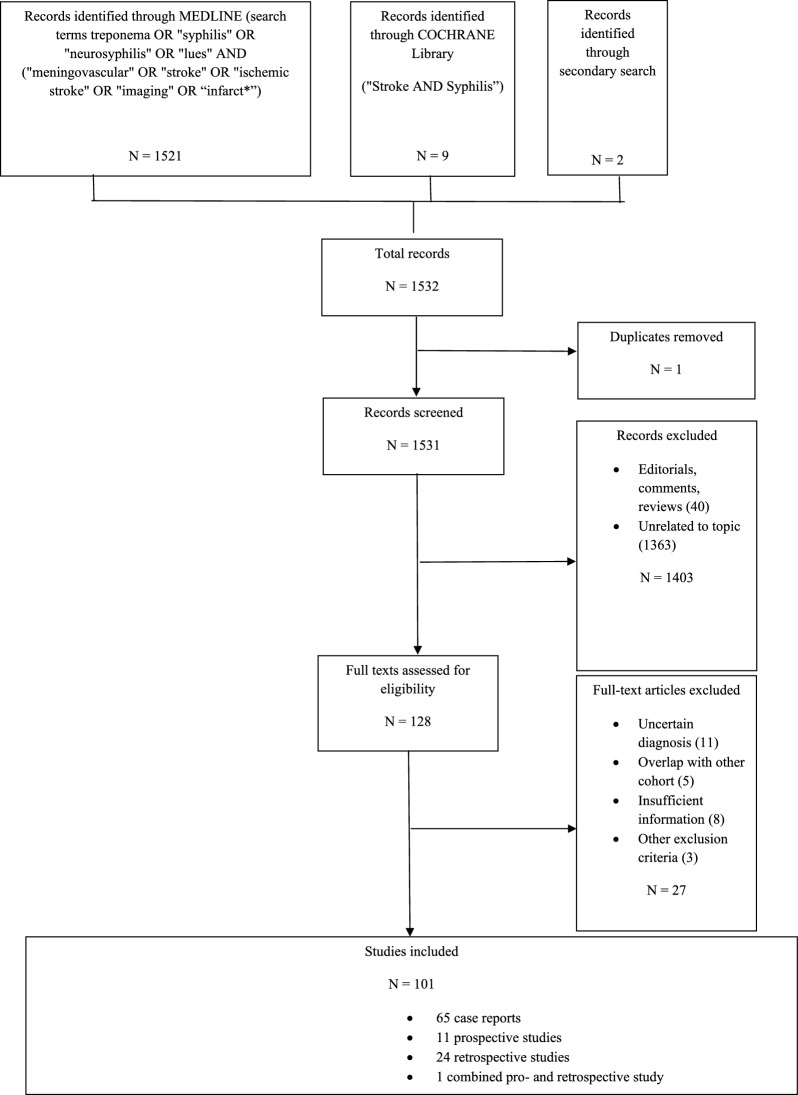
PRISMA flow chart depicting the results of the systematic literature research

A total of 1532 manuscripts on NS and stroke were found using the search terms. We screened a total of 1532 reports and included 101 into the final analysis (Fig. 2). We evaluated case reports separately from cohort studies and trials. The patient reported here was included in the analysis.

### Case reports

The literature revealed a total of 73 patients (67 male). Median age at diagnosis was 39 years (range 21 – 78 years).

**Fig. 3:**
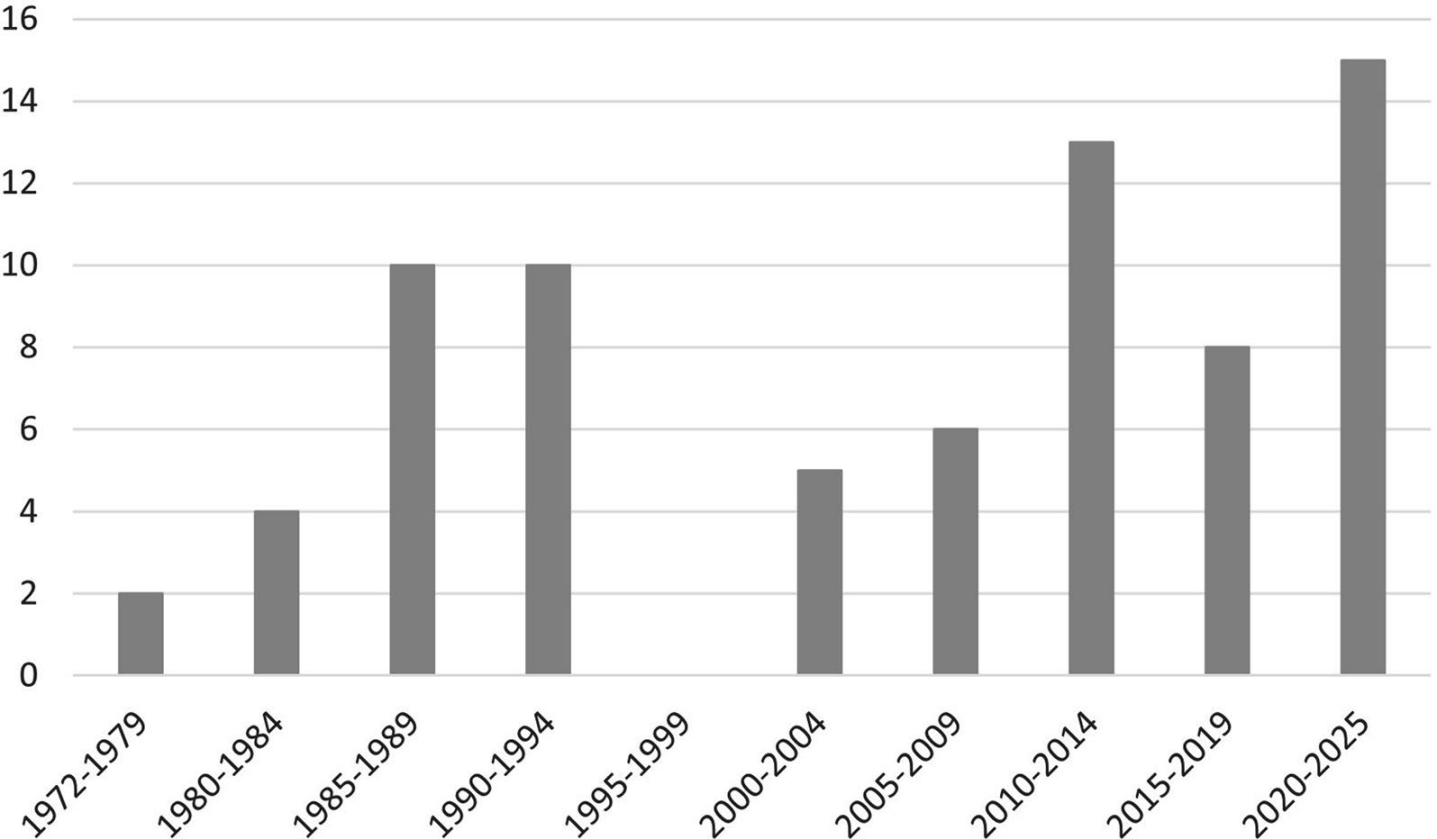
Temporal distribution of case reports as total number per decade

Case report publication dates of ranged from 1972 to 2025. Case numbers were at a low between 1995 and 1999 and peaked in the first half of the 2010s and again after 2020. For a graphic depiction of temporal distribution see Fig. 3.

Most reports were from the World Health Organization (WHO)Region of the Americas (AMRO; 29 patients) and from the European Region (EURO; 25 patients). 16 patients were from the Western Pacific Region (WPRO). The African Region (AFRO), the Eastern Mediterranean Region (EMRO), and the South-East Asia Region (SEARO) reported one patient each.

#### Stroke characteristics

Stroke was due to large-artery atherosclerosis and/ or stenosis in most (n = 39) patients, followed by small-vessel occlusion (n = 17 patients). Cardioembolic and hemorrhagic stroke were described in one patient and two patients respectively. Four patients had strokes of other determined etiology such as embolism caused by thrombi in the ascending aorta due to syphilitic aortitis. In eight patients, lack of data did not permit to classify the stroke. Two patients had competing etiologies.

In 59 patients, the stroke led to the diagnosis of syphilis. In 13 patients, the infection was diagnosed before. In one patient, this information was unavailable. Of the 13 patients with pre-diagnosed syphilis, two most probably had NS (headache and photophobia for two months; transient ischemic attack 6 years before). Only one patient developed the cerebrovascular event while on antibiotic treatment ([14]; patient with pre-diagnosed NS, cerebrovascular event five days after Penicillin G initiation). One patient had never received antibiotic treatment, the other patients presented with stroke four months to twenty years after antibiotic treatment for syphilis (information not available for one patient).

**Fig. 4:**
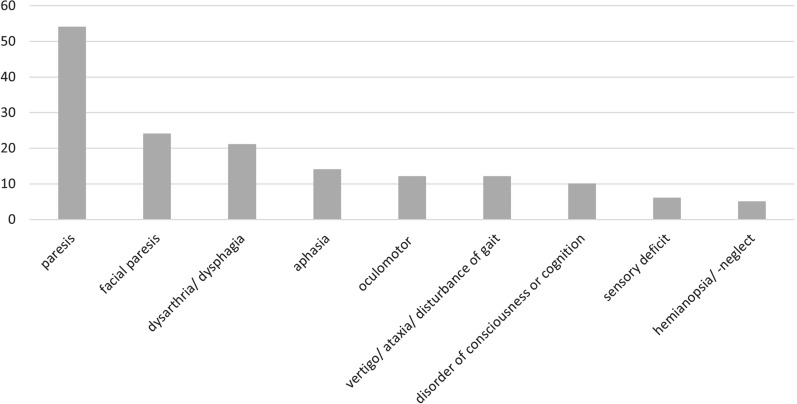
Presenting symptoms reported as total numbers. Multiple answers per patient were allowed

The median score on the National Institutes of Health Stroke Scale (NIHSS) at diagnosis was 4 (range 0—17). In six patients, insufficient information was available to determine the NIHSS. Motor and speech deficits were most frequently reported as presenting symptoms (see Fig. 4; multiple answers per patient were possible).

#### Results of paraclinical tests

CSF analysis was performed in 68 patients. Data on CSF cell count were available in 62 patients, for CSF cell differentiation in 40 patients, and for total CSF protein in 62 patients. The median cell count was 78 cells per µL (range 1–470). A median of 90% of cells were mononuclear (range 15–100). The median total protein was 116 mg/dl (range 31–2558).

Diagnosis of syphilis rested on serum and/ or CSF serological testing in 72 patients. In 66 out of 73 patients, at least one type of serological testing was performed in the CSF. In one patient, diagnosis was made on autopsy [15]. For 72/73 patients, results of nonspecific tests such as VDRL and RPR were reported. For 64 patients, results of at least one Treponema pallidum-specific antibody test in the serum and/ or CSF were available. Of the nine patients for whom no specific testing was reported, five had been published before 1990. CSF/serum antibody indices were reported for four patients: IgG index for two patients (2.0 and 2.11) and the TPHA ratio for two patients (5.2 and 12.8) at first available testing [16–19].

In the five patients who did not have CSF analysis, extracranial vascular pathology such as thrombi, stenosis and aneurysms due to arteritis of the aorta and the subclavian artery were diagnosed. In these patients, the diagnosis of NS cannot be made. The same is true for a patient with vasculitis of the common carotid artery, normal CSF cell count and negative CSF RPR [20] and a young man with extracranial large vessel pathology and negative VDRL in the CSF (cell count not reported; [21]). Two more patients did not meet our NS criteria: one had a sudden-onset brainstem syndrome and positive specific and nonspecific treponemal tests in the serum. However, CSF cell count and VDRL were normal/ negative, an MRI was not available [22]. Another patient with extracranial vessel pathology had a normal CSF cell count and no signs of intracerebral vascular disease on autopsy. Serology was positive in the serum, but not done/reported in the CSF [23]. One patient with hemorrhagic infarction and left internal jugular vein occlusion had a positive serum RPR [24]. However, CSF RPR as well as cell count, IgG index, and oligoclonal bands were all normal/negative.

In 49 patients, cerebral magnetic resonance imaging (MRI) was available. It showed supratentorial cortical infarct locations in 23 patients, supratentorial white matter or basal ganglia infarcts in 32 patients, and infratentorial infarcts in 33 patients (multiple answers were possible). In four patients, the infarct location was not reported; none of these had an MRI.

#### Therapeutic approach and outcome

Fifty-five patients were treated with penicillin, six with ceftriaxone. Two of these received both antibiotics. One patient was treated with tetracyclines. In 13 patients no antibiotic treatment was mentioned. Five of these died in the course of their disease.

Three patients received intravenous thrombolysis as part of their acute stroke treatment. In two of these patients, thrombectomy was additionally performed. One of these patients died due to re-occlusion of the vertebral and basilar arteries [25]. The other had a good outcome (mRS 1) [26].

For 18 patients, the use of antiplatelet agents was reported. Four patients received lipid-lowering agents as secondary stroke prophylaxis. One patient received warfarin due to a floating aortic thrombus with resultant systemic and cerebral embolisms [4].

Other therapeutic regimens included steroids and cyclophosphamide due to suspected vasculitis and antiretroviral therapy in patients diagnosed with HIV (n = 28).

Time to last follow-up was stated in 47 patients (median 180 days, range 4–1740). The outcome on the modified Rankin scale was detailed or could be estimated from the given data in 45 patients (median 2, IQR 0–6).

### Cohort studies and trials

We included 36 studies that investigated diverse patient cohorts for the prevalence and characteristics of stroke associated with NS (see Table 2). Date of recruitment ranged from 1974 to 2019 (start) and 1978 to 2023 (end). If the recruitment period was not stated, we specify the publication year in lieu.

**Table 2:** List of cohort studies and trials included in the systematic review

Author	Date of recruitment	Country	Primary cohort	n	Stroke + Syphilis (n)	Controls	Design	Outcome
[27]	1986	USA	Stroke/ TIA	218	2	Neurological patients w/o neurovascular disease (n = 150)	Prospective	T. pallidum seropositivity significantly higher in stroke group (χ^2^ = 7.7, p < 0.01)
[39]	1992	India	Neurosyphilis	7	4	None	Prospective	Descriptive
[40]	1974–1978	Denmark	Neurosyphilis	23	4	None	Retrospective	Descriptive
[41]	1975–1980	Korea	Neurosyphilis	24	5	None	Retrospective	Descriptive
[42]	1977–1980	Australia	Juvenile stroke (< 40yrs)	14	0	None	Retrospective	Descriptive
[43]	1984–1985	Zimbabwe	Stroke	91	8	None	Prospective	Descriptive
[44]	1984–1994	USA	Neurosyphilis	35	8	None	Retrospective	Descriptive
[45]	1988–1991	USA	Neurosyphilis, HIV positive	6	5	None	Retrospective	Descriptive
[46]	1990–1999	South Africa	Neurosyphilis	161	24	None	Retrospective	Descriptive
[47]	1992–1993	USA	Neurosyphilis, HIV positive	11	2	None	Prospective	Descriptive
[48]	1994–2019	Morocco	Neurosyphilis	178	16	None	Retrospective	Descriptive
[49]	1995–2010	Netherlands	First stroke/TIA	415	1	None	Mixed	Descriptive
[50]	1999–2006	India	Neurosyphilis	35	9	None	Retrospective	Descriptive
[51]	1999–2009	Germany	Nonatherosclerotic CNS vasculopathy	25	1	None	Retrospective	Descriptive
[28]	2000–2012	Taiwan	Syphilis	1585	61	Patients w/o syphilis (n = 6340)	Retrospective	Higher risk of ischaemic stroke in syphilis (adjusted HR 1.35; 95% CI, 1.01–1.80; p < 0.05)
[38]	2000–2015	Turkey	Neurosyphilis	141	13	None	Retrospective	Descriptive
[52]	2001–2002	Malawi	Stroke	98	0	None	Prospective	Descriptive
[53]	2001–2015	Morocco	Neurosyphilis	330	53	None	Retrospective	Descriptive
[54]	2002	Sudan	Stroke	96	4	None	Prospective	Descriptive
[55]	2002–2006	China	Neurosyphilis	14	6	None	Retrospective	Descriptive
[6]	2003–2014	USA	HIV	1599351	324	Internal controls	Retrospective	Stroke in HIV patients associated with neurosyphilis (OR4.38; 95% CI 21–5.97)
[56]	2005–2011	China	Neurosyphilis, HIV negative	149	21	Stroke patients w/o syphilis (n = 1570)	Retrospective	Descriptive
[57]	2005–2012	China	Neurosyphilis, HIV neg	149	36	None	Retrospective	Descriptive
[58]	2007–2009	Thailand	Stroke	284	7	None	Prospective	Descriptive
[36]	2007–2015; 2016 prospective	Australia	Neurosyphilis	25	2	None	Retrospective	Descriptive
[59]	2008–2010	India	Neurosyphilis	16	1	None	Prospective	Descriptive
[8]	2009–2019	China	Stroke, HIV positive, HAART-naive	105	36	Stroke-free HIV positive, HAART naive patients (n = 2762)	Retrospective	Syphilis is risk factor for stroke in this primary population (OR 2.003, 95% CI 1.300–3.089, p = 0.002)
[29]	2010–2015	Taiwan	Syphilis, HIV negative, no severe cerebrovascular disease	20601	1161	Patients w/o syphilis (n = 20601)	Retrospective	Syphilis is a risk factor for stroke in this primary population: ischaemic stroke (RR 68%, 95% CI 1.52–1.87, P < .001), haemorrhagic stroke (RR 114%, 95% CI 1.74–2.64,P < .001)
[60]	2011–2014	Portugal	Stroke	323	1	None	Retrospective	Descriptive
[31]	2012–2017	Brazil	First stroke	269	32	None	Prospective	Descriptive
[61]	2012–2018	China	Neurosyphilis, HIV neg	102	47	None	Retrospective	Descriptive
[62]	2012–2022	China	Neurosyphilis	50	18	None	Retrospective	Descriptive
[63]	2013–2018	China	Neurosyphilis, first stroke	179	179	Matched patients w/o syphilis	Prospective	Descriptive
[64]	2015–2016	Brazil	Stroke/ TIA	1119	53	None	Retrospective	Descriptive
[65]	2015–2023	China	Syphilis, HIV positive	60	18	None	Retrospective	Descriptive
[66]	2019–2020	Thailand	Stroke/ TIA	344	11	Patients with latent syphilis, neuroyphilis, w/o syphilis	Prospective	Descriptive

All studies combined included n = 1,626,633 patients in the primary cohorts (range 6–1,599,351) and n = 2173 patients with syphilis and stroke (range 0–1161). The studies included patients from all WHO regions: 446/36 (primary cohort/ meningovascular syphilis cohort) from AFRO, 1,601,009/426 from AMRO, 686/32 from SEARO, 927/20 from EURO, 508/69 from EMRO, and 23,057/1590 from WPRO.

Primary cohorts included hospitalized stroke patients (general/juvenile/first ever stroke; 11 studies), hospitalized HIV patients (± syphilis, 3 studies), NS patients (± HIV, ± first stroke; 19 studies), and patients diagnosed with nonatherosclerotic CNS vasculopathy (1 study).

In nine primary cohorts, HIV-status was explicitly specified (5 positive, 4 negative). Stroke was defined as either juvenile stroke in one cohort or first ever stroke in three cohorts. Twenty-nine cohorts were explicitly derived from a hospital setting.

Eight studies included control groups. The design was retrospective in 23, prospective in 11. Two studies combined retro- and prospective approaches. Quality was low to very low in almost all studies due to unclear or high risk of detection bias, small sample sizes, lack of control group and/or randomized design, high risk of selection bias, retrospective design and/or incomplete information on all or part of the cohort.

Thirteen studies reported the percentage of male patients (median 87%; range 40–100). Mean age at diagnosis in the NS group varied between 41 and 80 years (data available from eight trials with more than two patients).

While most studies were descriptive, statistical parameters were provided in five. These demonstrated higher seropositivity for T. pallidum in stroke patients compared to patients without neurovascular disease and a higher risk of ischemic stroke in patients with compared to those without syphilis [27, 28]. They also showed an association of stroke with NS in HIV patients, a higher risk of stroke in HIV-positive, HAART-naïve patients with compared to those without syphilis, and a higher risk of ischemic and hemorrhagic stroke in HIV negative patients without severe cerebrovascular disease if they had syphilis [6, 8, 29].

## Discussion

Syphilis is a risk factor for cerebrovascular events [28, 29]. Numbers of case reports have increased after 2010, although this may be due to increased awareness and general publication activity. There was no marked variation in the geographic distribution of manuscripts over the time we considered for this review. This suggests that the prevalence of NS remains steady or may be rising, even in high-income countries [30]. However, regional variation exist: in a retrospective investigation of all NS cases presenting to a maximum care hospital between 2020 and 2024 (Ludwig-Maximilians-University Munich; n = 17), no stroke cases were found. Contributing factors for a high NS prevalence include changes in sexual behavior following the introduction of efficient treatment of HIV.

Our patient suffered from meningovascular syphilis, compatible with early as well as late NS. NS usually manifests with cerebral ischaemia rather than hemorrhagic stroke, although the latter may occur as well. The presumed pathomechanism of stroke in NS is vasculitis of the small, medium, and large vessels of the CNS, causing vessel narrowing and occlusion. Endothelial inflammation may be mediated by treponemal proteins such as Tp0965 and Tp17, that have been shown to increase endothelial expression of adhesion molecules and vascular endothelial growth factor [2, 31].

Our patient presented with motor deficits that rank among the most common presenting symptoms in NS-associated stroke. This coincides with reports listing the middle cerebral artery territory and the brainstem as the most frequent stroke locations [3]. Alternatively, strokes in other locations may be underreported as symptoms tend to be more subtle. Particularly cognitive deficits are likely to be underreported.

In this review, we found a relatively equal distribution of supra- and infratentorial strokes as well as of cortical and white matter ischemia. Stroke etiology was most often large-vessel disease. The median NIHSS of the cohorts included in this review was 4. This is lower than in unselected stroke cohorts and may reflect the high proportion of microangiopathic pathology and the rare occurrence of cardioembolic stroke [32].

The male:female ratio reflects the overall predominance of syphilis in the male population. However, an increase of syphilis in women has been observed [1, 7]. The median age in NS was low compared to general stroke cohorts. This may reflect the higher NS prevalence in a sexually active population. The diagnosis of NS remains complicated which is reflected in the variability of diagnostic criteria worldwide and in the possible interpretation of the various laboratory tests. In most published cases, testing of serum and CSF is used to diagnose NS [1, 33]. CNS involvement may be deduced from CSF pleocytosis and/or elevated CSF protein, although normal CSF findings can occur in NS [2]. Treponemal tests with a high specificity confirm the diagnosis, while non-treponemal tests such as the Venereal Disease Research Laboratory (VDRL) test are used as markers of disease activity. T. pallidum PCR is more sensitive in early stages of the disease when a higher bacterial load is present, but strongly depends on the PCR technique and the specimen type (range: 3 – 100%) [34]. However, false negative and false positive diagnoses of NS occur due to limitations of these tests, e.g. limited sensitivity of the CSF VDRL (30 – 70%) [35–37].

The current CDC guidelines distinguish possible, likely and verified disease [13]. A reactive nontreponemal and treponemal test, consistent clinical symptoms and a reactive VDRL in the CSF are required to diagnose verified NS. Particularly older case reports often lack data that would allow for a definite classification according to the CDC, mostly for lack of CSF testing and determination of Treponema pallidum-specific antibodies. To account for the heterogeneity of diagnostic standards in different countries and time periods, we used more generous NS criteria. We identified ten patients who did not meet these criteria. These included predominantly patients with stroke due to extracranial vascular pathology.

The predominant antibiotic used to treat NS still is penicillin, which remains the therapy of choice. However, there is no indication that ceftriaxone is less efficient in NS than penicillin [1, 38]. It may thus be used as alternative treatment in patients known to be allergic to penicillin. Response to therapy should be verified by determining serum activity parameters such as VDRL. The outcome after sufficient antibiotic treatment is largely determined by residual stroke symptoms.

There are few cases published on thrombectomy and intravenous thrombolysis in NS. The little information there is does not provide evidence that NS patients should be treated differently from other stroke patients, e.g. due to higher bleeding rates. The heterogeneity of secondary prophylaxis in NS-associated stroke reflects the lack of specific guidelines. Furthermore, the use of antiplatelet agents and statins in this indication may be underreported in the case reports.

Challenges in the analysis of the literature on stroke in NS include the heterogeneity of patient cohorts and geographical and historical variations in diagnostic and therapeutic approaches. Furthermore, the extreme difference in included patients per study introduces a bias. Few prospective controlled trials exist. Due to the lack of high-quality individual data, no meta-analysis could be performed.

## Conclusion

NS prevalence is on the rise and should be considered as a cause of stroke, particularly in young patients without conventional vascular risk factors. Comorbidity with HIV should be actively sought and treated if present. Penicillin and ceftriaxone remain the mainstay of syphilis treatment.

## Data Availability

Data are available upon reasonable request from the corresponding author.
